# Study on the Structural, Optical, and Magneto-Optical Properties of Bi_2_O_3_-Pb_3_(BO_3_)_2_-Ga_2_O_3_-PbO Glasses for Temperature-Insensitive Magneto-Optical Isolator Applications

**DOI:** 10.3390/ma18204750

**Published:** 2025-10-16

**Authors:** Rui Wan, Chen Guo, Hang Jiang, Yong Jiang, Xianda Li, Yongmao Guan, Pengfei Wang

**Affiliations:** 1 State Key Laboratory of Ultrafast Optical Science and Technology, Xi’an Institute of Optics and Precision Mechanics, Chinese Academy of Sciences (CAS), Xi’an 710119, China; wanrui@opt.ac.cn (R.W.); guochen@opt.cn (C.G.); bonfiretank@163.com (X.L.); guanyongmao20@mails.ucas.ac.cn (Y.G.); 2 Center of Materials Science and Optoelectronics Engineering, University of Chinese Academy of Sciences, Beijing 100049, China; 3 Southwest Institute of Applied Magnetics, Mianyang 621006, China; jianghang998@163.com; 4 School of Mathematics and Physics, Southwest University of Science and Technology, Mianyang 621010, China; y_jiang@swust.edu.cn

**Keywords:** bismuthate glass, Verdet constant, laser-induced damage threshold, glass network

## Abstract

In this work, bismuthate glasses with compositions of 64Bi_2_O_3_-(25-x)Pb_3_(BO_3_)_2_-11Ga_2_O_3_-xPbO (where x = 2, 7, 12, 17) were prepared by the melt-quenching method, and their density, thermodynamic stability, Raman spectra, X-ray photoelectron spectra, Verdet constant, and nanosecond laser-induced damage threshold (LIDT) were characterized. As the content of PbO increases, the thermodynamic stability and laser-induced damage threshold of the glass gradually decrease, which corresponds to the increase in the glass’s optical basicity, the rise in non-bridging oxygen content, and the valence state transition of Bi ions observed in structural studies. A relatively large Verdet constant was obtained in the glass with the composition of 64Bi_2_O_3_-8Pb_3_(BO_3_)_2_-11Ga_2_O_3_-17PbO, with a value of −0.191 min·G^−1^·cm^−1^ at a wavelength of 633 nm, which is much larger than that of commercially diamagnetic glasses. In addition, the variation in the Verdet constant at 1064 nm between 20 and 80 °C is less than 0.4 × 10^−5^ K^−1^, which indicates that these bismuthate glasses are good candidates for magneto-optical devices under thermally unstable conditions.

## 1. Introduction

As one of the greatest inventions of the 20th century, lasers possess advantages that ordinary light sources lack, such as excellent directionality, high brightness, monochromaticity, and coherence [1]. They are widely applied in numerous fields, including industry [2], communication [3], medicine [4], and military [5]. During the operation of high-power lasers, high-energy reflected light can cause damage to front-end systems (such as resonant cavities and seed sources), posing a serious threat to the reliability of the laser system. Therefore, a method capable of effectively isolating reflected light is required [6].

Magneto-optical isolators, as “passive and non-reciprocal” optical devices, are essential components in high-power laser systems to prevent optical feedback from damaging lasers and other optical elements [7]. A magneto-optical isolator consists of a Faraday rotator, polarizers, and a magnetic ring. The Faraday rotator, a key component of the isolator, is composed of magneto-optical materials exhibiting the Faraday effect and magnets providing a constant magnetic field. To meet the isolation requirements of magneto-optical isolators, such magneto-optical materials need to not only have a high Verdet constant to shorten the element length and a high laser-induced damage threshold (LIDT) to prevent damage of optical elements, but also a low Verdet constant temperature coefficient to effectively suppress the depolarization phenomenon caused by temperature differences [8].

Currently, magneto-optical isolators used in visible light and 1.0 μm wavelength laser systems generally employ rare earth (RE)-doped paramagnetic magneto-optical materials with large Verdet constants and low optical absorption coefficients, such as terbium gallium garnet (TGG) crystals and terbium-doped glasses [8,9]. Compared with magneto-optical crystals, which are challenging to produce in large sizes and at high optical quality, as well as being costly, magneto-optical glasses offer advantages such as a widely adjustable range of compositions, high RE solubility, large size, and low absorption coefficient [10]. These characteristics have led to extensive research on optical glasses by numerous researchers in the application fields of magneto-optical functional devices. Although terbium-containing paramagnetic magneto-optical materials exhibit large Verdet constants, abrupt and obvious temperature changes can cause significant drifts in the magneto-optical coefficients and deflection angles of paramagnetic media, ultimately leading to the failure of magneto-optical isolators in work [8].

Diamagnetic glasses containing ions with high polarizability, such as As^3+^, Te^4+^, Bi^3+^, Pb^4+^, Sb^3+^, and In^3+^, are an important branch in the field of magneto-optical materials greatly different from their RE-doped paramagnetic counterparts [11]. Since these diamagnetic ions have no permanent electronic orbital magnetic moment, they can generate an induced magnetic moment opposite to the direction of the applied magnetic field under the action of a magnetic field. Compared with RE ions that exhibit paramagnetic properties, these diamagnetic ions mostly act as network intermediates in glass compositions. When their content is sufficiently high, they can form part of the network structure together with glass formers. Therefore, the magneto-optical properties of diamagnetic glasses are closely related to the glass network structure, which renders the Verdet constant of diamagnetic magneto-optical glasses temperature-insensitive [12]. High-performance diamagnetic glasses’ theoretical Verdet constant temperature coefficient is less than 1 × 10^−3^ K^−1^, endowing them with great application potential in harsh application environments and characteristic scenarios with large temperature variations in contrast to constant-temperature laboratory conditions [13].

At present, studies have been conducted on a series of diamagnetic glass systems, such as silicate, borate, chalcogenide, and tellurite glasses. Corning Incorporated found in its research on the diamagnetic properties of oxide optical glasses that the Verdet constant increases linearly with the rise in the molar content of PbO in silicate and borate glass systems [14]. For the 20TeO_2_-80PbO composition, the highest diamagnetic Verdet constant was achieved, which is 0.15 min·G^−1^·cm^−1^ and 0.05 min·G^−1^·cm^−1^ at 700 nm and 1060 nm, respectively [14]. Ovcharenko et al. tested the magneto-optical properties of the TeO_2_-WO_3_-Bi_2_O_3_ and TeO_2_-WO_3_-PbO glass systems. The results show that the glasses in these systems exhibit diamagnetism, with their Verdet constants ranging from 0.08 to 0.11 min·G^−1^·cm^−1^ [15]. Xu et al. systematically investigated the diamagnetic properties of chalcogenide glasses in the GeS_2_-Ga_2_S_3_ and GeS_2_-Ga_2_S_3_-PbI_2_ systems. The maximum Verdet constant of 0.230 min·G^−1^·cm^−1^ at 633 nm was measured in the glass with the composition of 68GeS_2_-17Ga_2_S_3_-15PbI_2_ [16]. However, the prepared PbI_2_-doped chalcogenide glasses still maintain very high absorption coefficients near 1.0 μm, compared to commercial magneto-optical media. The temperature dependence of the Verdet constant in diamagnetic materials has also been investigated. Studies by Tan et al. showed that the Verdet constant of fused silica glass can be regarded as a constant over a wide temperature range from 25 °C to 400 °C [17]. Williams et al. found in their study that when the temperature of SF57 glass increases from 12 °C to 90 °C, the variation in the Verdet constant (ΔV/V) is only 1.29 × 10^−4^ K^−1^, which confirms that the Verdet constant exhibits excellent temperature stability in diamagnetic materials [18].

The most significant practical drawback of existing diamagnetic glasses is their relatively small Verdet constant. Therefore, there is an urgent need to develop novel high-performance diamagnetic glass materials. Compared with the aforementioned tellurite and lead glasses, bismuthate glasses not only have significant advantages in aspects such as physicochemical stability, thermal stability, and laser damage resistance, but also exhibit merits including a relatively higher Verdet constant, a lower temperature coefficient of their Verdet constant, and a wide mid-infrared transmission range [19]. As a result, they are potential magneto-optical dielectric materials for manufacturing high-performance diamagnetic magneto-optical isolators used in high-energy laser systems and have attracted great attention from researchers, albeit with great challenges too.

In this investigation, Bi_2_O_3_-Pb_3_(BO_3_)_2_-Ga_2_O_3_-PbO bismuthate glasses were fabricated using the melt-quenching technique. The physical, optical, and structure properties of glass samples, including density, thermodynamic stability, Raman spectra, X-ray photoelectron spectra, Verdet constant, and nanosecond laser-induced damage threshold (LIDT) were studied systematically. The studies showed that the novel bismuthate glasses exhibit a relatively high Verdet constant (−0.056 min·G^−1^·cm^−1^ @ 1064 nm), an extremely low Verdet constant temperature coefficient (<0.4 × 10^−5^ K^−1^), and a high laser-induced damage threshold (>8.33 J/cm^2^@1064 nm), thus demonstrating their promising application prospects in the field of temperature-insensitive magneto-optical isolators for high-power laser systems.

## 2. Materials and Method

### 2.1. Preparation of Bismuthate Glasses

Bismuthate glasses with a molar composition of 64Bi_2_O_3_-(25-x)Pb_3_(BO_3_)_2_-11Ga_2_O_3_-xPbO (where x = 2, 7, 12, 17), corresponding to sample numbers BP-1, BP-2, BP-3, and BP-4, respectively, were prepared using the conventional melt-quenching technique. The source information of raw materials is shown in Table 1. All high-purity commercial raw materials were accurately weighed (50 g total) according to the designed composition ratio, and then placed into an alumina crucible preheated to 950 °C for melting at this temperature for 40 min, with stirring performed every 20 min during the melting process. Subsequently, the glass melt was subjected to controlled cooling to 820 °C, held at this temperature for 10 min, and then poured into a brass mold preheated to 250 °C for shaping. Finally, the solidified glass was transferred to an annealing furnace, where it was held at 310 °C for 6 h before being slowly cooled down to room temperature to remove inside thermal stress. The obtained glass was cut into specimens with dimensions of 20 × 20 × 5 mm and Φ 20 × 5 mm, followed by precision optical polishing to meet the requirements of various performance tests. To suppress the reduction in heavy metal ions, ultra-dry oxygen was continuously and stably introduced into the inner chamber of the melting furnace throughout the entire melting process, with its flow rate consistently set at 0.3 L/min.

### 2.2. Characterization of the Bismuthate Glasses

**Figure 1:**
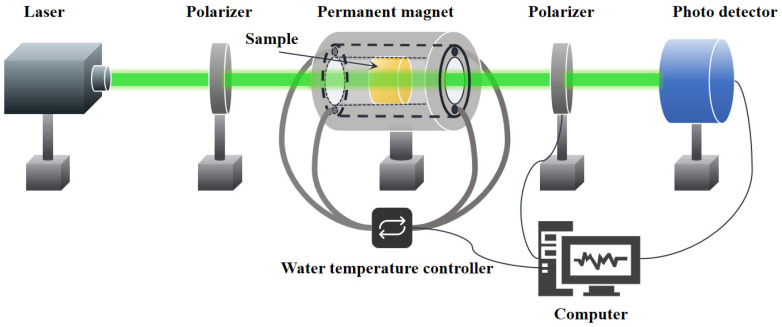
Schematic diagram of the variable-temperature Verdet constant testing platform.

The density of samples was measured based on Archimedes’ principle, using deionized water at 25 °C as the immersion liquid. The transmission spectra of the glass in the visible-to-infrared range were collected using a UV/Vis/NIR spectrophotometer (Jasco, V-570, Tokyo, Japan) and a Fourier-transform infrared (FTIR) spectrometer (Bruker, Vertex 70, Ettlingen, Germany), respectively. The refractive index of the bismuthate glass at 587.6 nm was measured using a V-prism refractometer (INESA, WYV-S, Shanghai, China). In addition, the Raman spectra and X-ray photoelectron spectroscopy (XPS) of the samples were measured using an HR Evolution laser Raman spectrometer (Horiba, LabRAM Soleil, Kyoto, Japan) with an excitation wavelength of 532 nm and an X-ray Photoelectron Spectrometer (Thermo Fisher, ESCALAB 250XI, Waltham, MA, USA), respectively, to evaluate the structural changes in the glass network. The magnetic moment and hysteresis loop of the samples were measured using a Superconducting quantum interference device‌ (Quantum Design, MPMS3, San Diego, CA, USA). The thermal properties of samples were analyzed using a differential scanning calorimeter (NETZSCH, DSC 404F1, Selb, Germany), with a heating rate of 10 °C/min under an air atmosphere. The laser-induced damage threshold (LIDT) of all glass samples was measured using the R-on-1 method with a 1064 nm laser (pulse width: 8 ns; repetition rate: 10 Hz; spot size: 0.32 mm; M^2^: 1.2). The damage criterion follows ISO 21254 [20], and the detailed procedures can be found in the study by Yu et al. [21]. The Verdet constants of the samples were measured using a home-made optical setup as shown in Figure 1, which consists of semiconductor lasers (Aunion, BP-E1 Series, Shanghai, China) at 532, 633, 808, and 1064 nm, a polarizer (DaHeng Optics, GCL-0510, Beijing, China) and an analyzer (Thorlabs, PRM1(/M)Z7, Newton, MA, USA), a photo detector (Thorlabs, PM100 D, Newton, MA, USA), a fixture with an embedded water channel, and a hollow cylindrical permanent magnet. Gaussmeter (AlphaLab, VGM, Salt Lake City, UT, USA) was employed to measure that the central region can generate a uniform axial magnetic field of 0.98 × 10^4^ Gauss. All tests were conducted at room temperature.

## 3. Results and Discussion

### 3.1. Physical Properties and Laser-Induced Damage Threshold

The physical properties of the bismuthate glasses, including density (ρ), refractive index (n), dielectric constant (ε), optical basicity (Λ_th_), oxide ion polarizability (α_O^2−^_), molar refractivity (R_m_), molar volume (V_m_), and oxygen packing density (OPD), were calculated using the formulae provided in the literature [22,23,24] and are listed in Table 2. As Pb_3_(BO_3_)_2_ in the glass is gradually replaced by PbO, all the aforementioned physical parameters of the glass increase progressively, except for the oxygen packing density (OPD). In general, the molar refractive index reflects the deformability of electron clouds in an external electric field [25,26]. Since the polarizability of Pb^2+^ and Pb^4+^ is much higher than that of B^3+^, the refractive index and R_m_ of the glass increase rapidly as the boron content decreases. The optical basicity (Λ_th_) of glass not only reflects the acid–base property inside the glass but also enables the determination of changes in the glass network structure from a microstructural perspective. As the polarizability of metal cations increases, oxygen ions become more prone to polarization, the optical basicity of the glass increases, and the binding of oxygen ions to their electron clouds weakens. At the macroscopic level, when the samples exhibit high optical basicity, the glass network gradually loosens due to polarization, ultimately leading to a decrease in glass stability.

**Figure 2:**
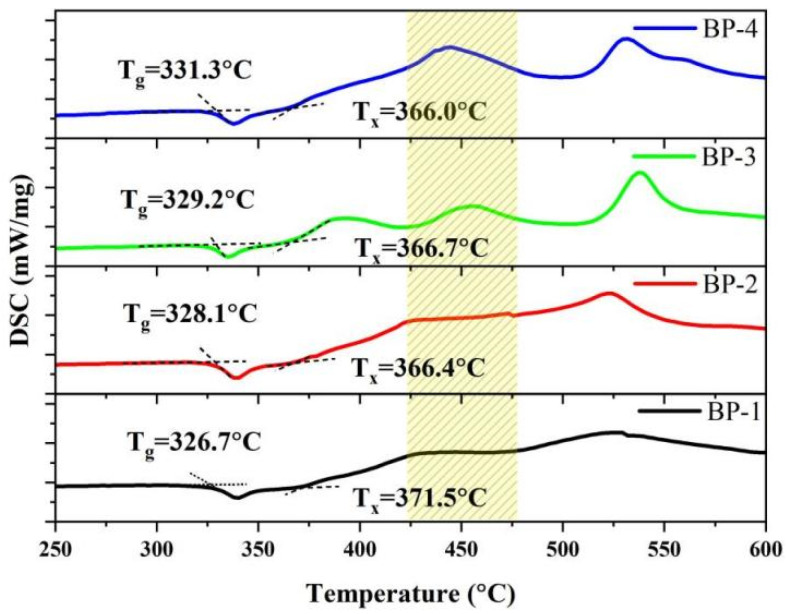
DSC curves of the bismuthate glasses.

To verify the changes in the macroscopic stability of the glasses, we obtained the thermodynamic characteristic temperatures of the series of bismuthate glasses using a differential scanning calorimeter (DSC), as shown in Figure 2. The glass transition temperature (T_g_), onset crystallization temperature (T_x_), and thermal stability parameter (ΔT = T_x_ − T_g_) of optical glass are crucial thermal performance indicators that indirectly measure the microscopic chemical bond strength, as well as the macroscopic thermal shock resistance and thermal processing performance of the glass [27]. As Pb_3_(BO_3_)_2_ is gradually replaced by PbO, the T_g_ of the glass increases gradually from 326.7 °C to 331.3 °C, while the T_x_ decreases from 371.5 °C to 366.0 °C. The thermal stability parameter of the glass gradually decreases from 44.8 °C to 34.7 °C, which is consistent with the variation trend of the physical parameters. Meanwhile, as the crystallization tendency of the glass intensifies, a new exothermic peak appears near 450 °C. This indicates that a further increase in PbO content leads to a change in the type of crystals precipitated from the glass.

**Figure 3:**
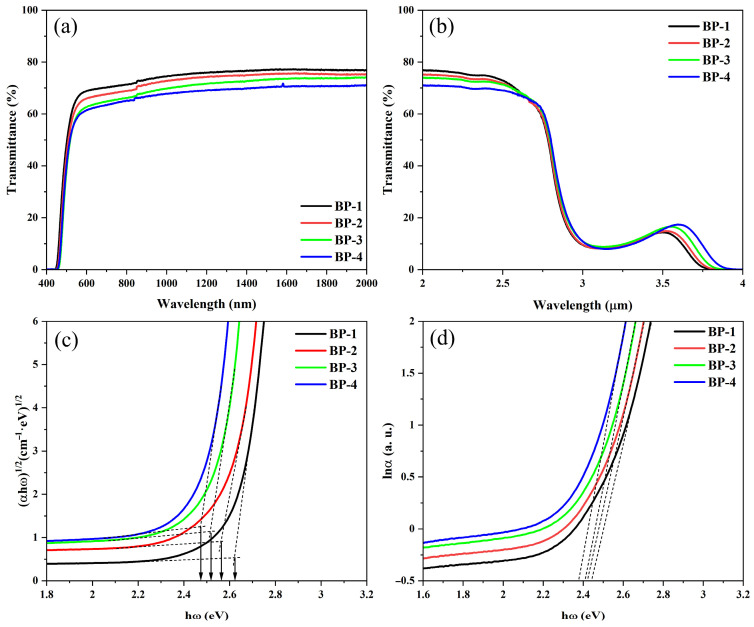
(**a**) Transmission spectra in the range of 400–2000 nm. (**b**) FTIR transmission spectra. Relationship between (**c**) (αhω)^1/2^ and hω, and (**d**) lnα and hω of the bismuthate glasses.

The spectral properties of the series of glasses are shown in Figure 3; all glass samples exhibit high transparency in the visible and near-infrared regions, except for an absorption peak caused by hydroxyl groups (OH^−^) in the range of 2.9–3.5 μm [28]. Among these samples, the BP-1 glass with a thickness of 5 mm achieves a maximum transmittance of 77%. As Pb_3_(BO_3_)_2_ in the series of samples is gradually replaced by PbO, the refractive index of the glass increases incrementally, leading to a gradual rise in the Fresnel reflection of the glass. Consequently, the overall transmittance of the glass decreases progressively. Meanwhile, as the content of B_2_O_3_ in the glass gradually decreases, the maximum phonon energy of the glass decreases incrementally, which broadens the infrared transmission range of the glasses. This can be confirmed by the slight red shift in the samples’ infrared cut-off wavelength.

The ultraviolet (UV) cut-off edge consists of two components: the strong absorption of the material and the Urbach tail absorption. The strong absorption is caused by inter-band transitions, which occur when electrons inside the material gain energy greater than the optical band gap. In contrast, Urbach tail absorption results from transitions between tail states formed at the top of the valence band and the bottom of the conduction band, which is due to the disordered structure of the glass. The optical band gaps can be estimated from the ultraviolet cut-off edge using a “proper extrapolation” method provided in the references [27,29]. As the component Pb_3_(BO_3_)_2_ is gradually replaced by PbO, the optical band gaps of the bismuthate glass series are estimated to be 2.62 eV, 2.56 eV, 2.52 eV, and 2.48 eV, respectively, showing a monotonically decreasing trend. This indicates that defects such as dangling bonds and vacancies in the glass network, as well as the energy level structure, have undergone changes [30]. With hω as the *X*-axis and ln α as the *Y*-axis, the reciprocals of the slopes of the linear parts of the curves were calculated to obtain the Urbach energies of the series of glasses, which are 0.093 eV, 0.105 eV, 0.114 eV, and 0.121 eV, respectively, also gradually increase with the increase in PbO content. This indicates that the increase in defect content in the glass leads to a higher degree of disorder in the glass network structure [27,31].

The LIDT characteristics of the samples were assessed using the R-on-1 testing mode. As Pb_3_(BO_3_)_2_ is gradually replaced by PbO, the LIDT values of the samples are 12.61 J/cm^2^, 11.27 J/cm^2^, 9.23 J/cm^2^, and 8.33 J/cm^2^, respectively, showing a gradual downward trend. At a low number of pulses, the heat accumulated from multiple pulses can cause damage to the material surface. As the PbO content increases, both the glass transition temperature and the glass stability of the glass decrease, which in turn leads to a reduction in the glass’s laser damage resistance. Even so, these glasses still meet the requirements for Verdet constant testing.

### 3.2. Structural Properties

**Figure 4:**
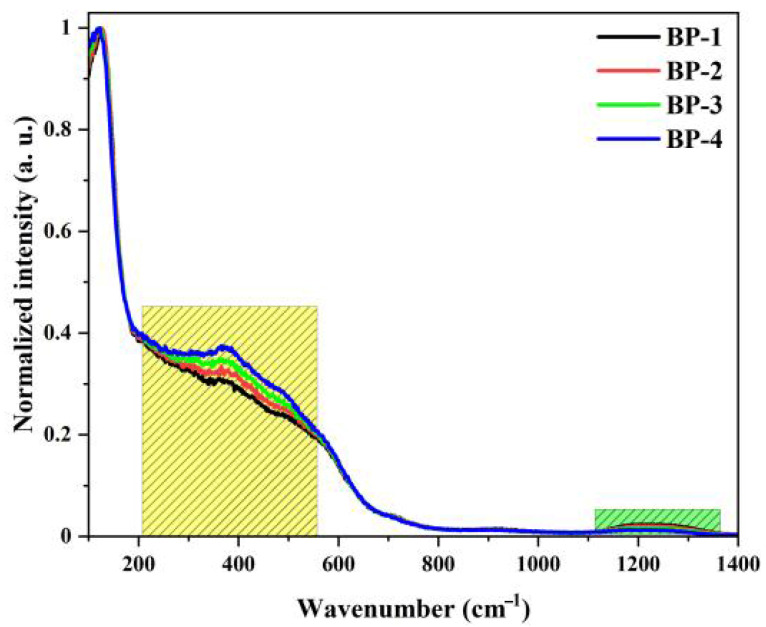
Raman spectra of the bismuthate glasses.

To analyze the structural changes in the glasses, Raman spectroscopy was used for characterization. Figure 4 shows the normalized Raman spectra of this series of glasses, which mainly consist of three vibration bands with their central peak positions at 114 cm^−1^, 370 cm^−1^, and 1220 cm^−1^. The vibration band at 114 cm^−1^ is attributed to the vibration of Pb-O covalent bonds and the Bi-O vibrations in bismuth oxide polyhedra. The vibration bands near 370 cm^−1^ and 1220 cm^−1^ are mainly associated with the Bi-O-Bi stretching vibration of [BiO_6_] octahedral units, the vibration of [BO_4_] tetrahedra, and the B-O-B stretching vibration of [BO_3_] pyramids, respectively [32]. As Pb_3_(BO_3_)_2_ is gradually replaced by PbO, the intensity of the vibration band at 370 cm^−1^ increases gradually, while that at 1220 cm^−1^ decreases. This is attributed to the gradual decrease in the content of B_2_O_3_ in the glass network, which leads to a gradual reduction in the proportion of B-O-B linkages during the formation of the glass network and a gradual increase in the content of [BiO_6_] octahedra.

**Figure 5:**
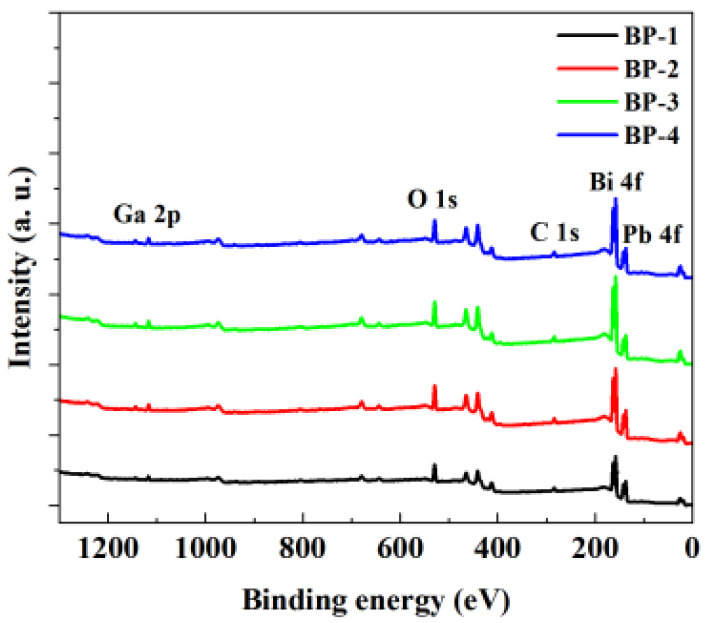
X-ray photoelectron spectroscopy of the bismuthate glasses.

X-ray photoelectron spectroscopy (XPS), when combined with Raman spectroscopy, serves as a powerful tool to determine the effect of component variations on the structure of magneto-optical glasses and explore the intrinsic mechanism underlying the changes in the magneto-optical properties of glass samples. The XPS survey spectra in Figure 5 shows the presence of abundant Bi 4f, Pb 4f, O 1s, and Ga 2p peaks in the glasses. It can be observed that the signals of Bi 4f and Pb 4f increase as Pb_3_(BO_3_)_2_ is gradually replaced by PbO.

**Figure 6:**
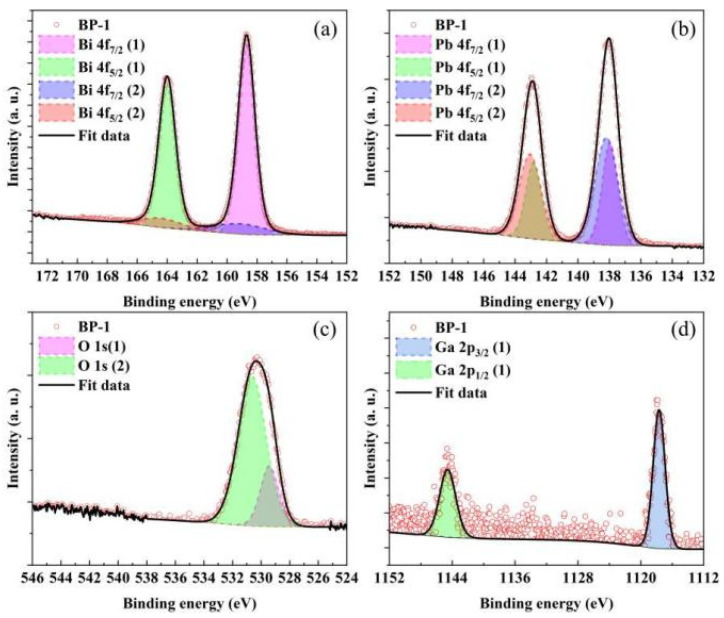
Deconvolution high-resolution XPS profiles of (**a**) Bi 4f, (**b**) Pb 4f, (**c**) O 1s, and (**d**) Ga 2p core-level spectra of BP-1 glass.

**Figure 7:**
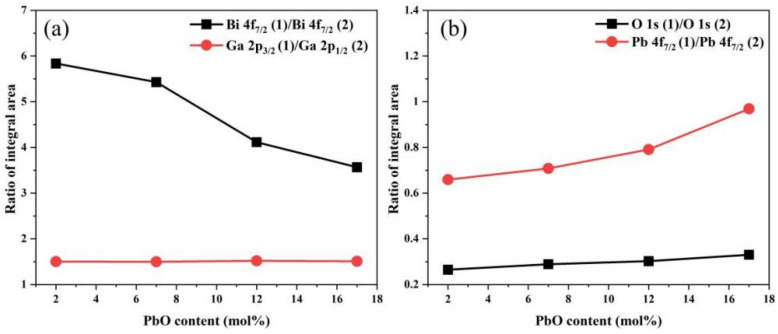
Variation in the high-resolution XPS integral area ratio of (**a**) Bi, Ga and (**b**) O, Pb as a function of PbO content.

Asymmetry was observed on the higher binding energy side of the main peaks in the high-resolution XPS profiles of Bi 4f, Pb 4f, and O 1s for all samples. Therefore, high-resolution XPS profiles of all samples were subjected to Gaussian–Lorentz function fitting to conduct a more in-depth analysis of the structural changes in the glasses. Figure 6 and Table 3 show the fitted spectrum of the BP-1 glass and the fitting results of all investigated glass samples, respectively, while the variation in the integrated area ratio of high-resolution XPS spectra with PbO content is presented in Figure 7.

In the O 1s core-level spectra of all analyzed samples, two distinct deconvoluted Gaussian–Lorentzian peaks are observed, corresponding to two different types of oxygen bonds [33,34]. In general, the O 1s (1) peak with a lower binding energy (BE) (529.4 eV) corresponds to non-bridging oxygen bonds (NBO), while the O 1s (2) peak with a higher BE (530.6 eV) corresponds to bridging oxygen bonds (BO). The increase in the ratio of non-bridging oxygen bonds (NBO) to bridging oxygen bonds (BO) with the rise in PbO content indicates a gradual increase in non-bridging oxygen (NBO) within the glass network, which leads to a deterioration in the connectivity of the glass network. Macroscopically, this results in a decrease in the stability of the glass, which is consistent with the thermodynamic test results of the glasses. Bi atoms mainly exist in the form of [BiO_6_] octahedra in bismuthate glass. However, due to electron loss caused by the strong repulsive force between certain oxygen ions in the glass network and the 6s^2^ electron orbitals of Bi^3+^, Bi can exhibit two valence states: +3 and +5. The Bi 4f peak was deconvoluted into four peaks, which correspond to the spin–orbit split peaks of 4f_7/2_ and 4f_5/2_ for Bi^3+^ and Bi^5+^, respectively. Among these peaks, the one labeled (1) has a lower BE (158.7 eV) and corresponds to Bi^3+^, while the one labeled (2) has a higher BE (159.1 eV) and corresponds to Bi^5+^. As the content of PbO increases, the area ratio of the Bi 4f_7/2_ (1) peak to the Bi 4f_7/2_ (2) peak decreases from 5.83 to 3.57, which indicates that more Bi^3+^ are converted to Bi^5+^ [33,35]. Pb atoms mainly exist in the form of [PbO_4_] square pyramids. Due to the characteristic of their asymmetric coordination, the Pb 4f peak was deconvoluted into four peaks, which correspond to the spin–orbit split peaks of 4f_7/2_ and 4f_5/2_ for Pb^0^ and Pb^4+^, respectively. The one labeled (1) has a lower BE (138.0 eV) and corresponds to Pb^0^, while the one labeled (2) has a higher BE (138.2 eV) and corresponds to Pb^4+^. The area ratio of the Pb 4f_7/2_ (1) peak to the Pb 4f_7/2_ (2) peak increases from 0.66 to 0.97, which indicates that the increase in PbO composition in the glass converts more Pb^4+^ to Pb^0^ [36,37]. The Ga 2p peak was deconvoluted into two peaks, which correspond to the spin–orbit split peaks of Ga 2p_1/2_ (1144.6 eV) and Ga 2p_3/2_ (1117.7 eV) orbitals, respectively. It can be observed that the binding energy of the Ga 2p_3/2_ peak does not exhibit a significant chemical shift with the increase in PbO content. This indicates that in this series of samples, Ga exists stably in the form of a [GaO_4_] tetrahedral structure [35,38].

### 3.3. Magnetic Properties and Verdet Constant

**Figure 8:**
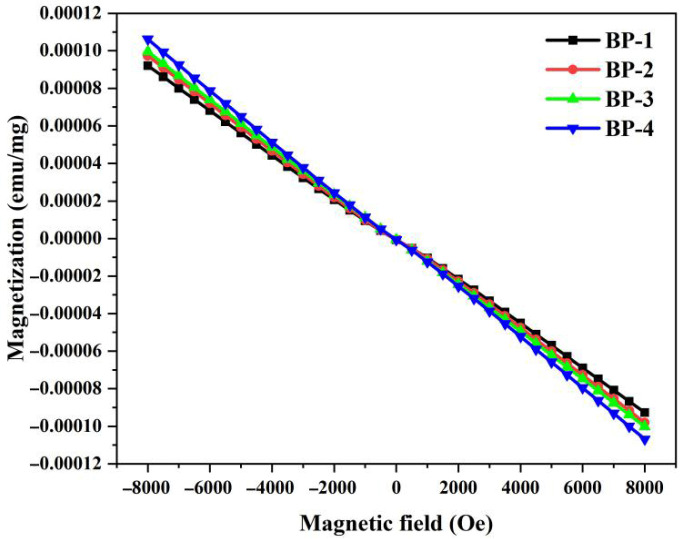
Magnetization of bismuthate glasses versus applied magnetic field.

Figure 8 shows the hysteresis loops of samples measured at room temperature. All these investigated bismuthate glass samples exhibit typical diamagnetic characteristics, and no magnetic susceptibility loops are observed. As Pb_3_(BO_3_)_2_ is gradually replaced by PbO, the magnetization of the samples increases progressively. Magnetic susceptibility (χ) is an indicator used to measure the ease with which glass materials can be magnetized by a magnetic field. Based on the hysteresis loops of the samples, with the increase in the number of samples and the content of lead monoxide (PbO), the magnetic susceptibilities of the glasses are −11.55 × 10^−6^ emu/g, −12.21 × 10^−6^ emu/g, −12.49 × 10^−6^ emu/g, and −13.32 × 10^−6^ emu/g, respectively. The magnetic properties of any material originate from the magnetic moments of individual atoms and the change in orbital momentum induced by an external magnetic field contributes to diamagnetism [39]. Diamagnetism is proportional to both the number of electrons and the square of the orbital radius of closed electron shells [40]. In the BP series glasses, bismuth mainly exists in the valence states of Bi^3+^ and Bi^5+^. The electron configurations of both valence states have no unpaired electrons, so both exhibit diamagnetism. Similarly, lead, gallium, and boron also exhibit diamagnetism. The Pb^2+^ ion has two electrons in its outer shell, corresponding to an electron configuration of 6s^2^. Consequently, it exhibits a strong s^2^-sp electronic transition amplitude, involving transitions from the ^1^S_0_ energy level to the ^1^P_1_, ^3^P_0_, ^3^P_1_, and ^3^P_2_ energy levels. Studies have shown that in certain types of oxide glass systems, such transitions result in a relatively strong diamagnetism [14]. According to Pascal’s method and the theoretical magnetic susceptibility of diamagnetic ions, the order of the contribution of each cation to diamagnetism in BP glasses is χ_Pb_ > χ_Bi_ > χ_Ga_> χ_B_ [24]. Therefore, as the content of B_2_O_3_ in the glass gradually decreases, the diamagnetic susceptibility of the samples gradually increases.

**Figure 9:**
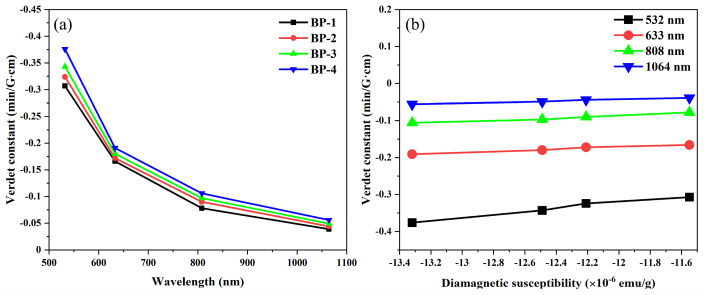
Dispersion property of Verdet constant with (**a**) wavelength and (**b**) diamagnetic susceptibility for bismuthate glasses.

The Verdet constants of the samples at different wavelengths are presented in Figure 9 and Table 4. To verify the reliability of the system, we selected fused silica glass as a standard sample. Its Verdet constant at a wavelength of 633 nm is −0.013 min·G^−1^·cm^−1^, which is essentially consistent with the results reported in reference [41]. According to previous studies, the Verdet constant is often proportional to the refractive index of glass [42]. For the BP series glasses, the replacement of B^3+^ by Pb^2+^ leads to a gradual increase in the glass’s optical basicity, oxygen ion polarizability, and refractive index, which ultimately results in a gradual increase in the glass’s Verdet constant. As shown in Table 5, under the condition of ensuring the stability of the glass, the Verdet constant of BP-4 glass at 633 nm can reach −0.191 min·G^−1^·cm^−1^, which is higher than some diamagnetic oxide glass. Furthermore, it is found that the Verdet constants at various wavelengths exhibit almost a linear relationship with the diamagnetic susceptibility of the studied glass. This implies that the Verdet constant of the glass can be directionally enhanced by introducing more components with high diamagnetic susceptibility into the glass.

**Figure 10:**
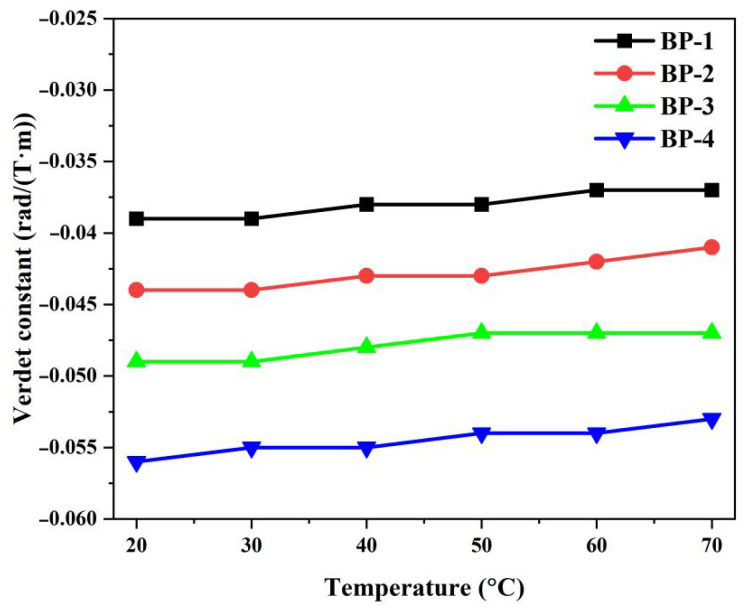
Temperature dependences of the Verdet constant of the bismuthate glass samples at 1064 nm.

To clarify the temperature dependence, the Verdet constants of the glass samples at 1064 nm were measured under different temperatures; the results are presented in Figure 10 and Table 6. It can be observed that when the temperature increases from 20 °C to 70 °C, the Verdet constant of the bismuthate glass remains almost unchanged, and its slope coefficient is less than 0.4 × 10^−5^ K^−1^ at 1064 nm. Compared with Tb-doped aluminosilicate glass (−8.9 × 10^−4^ K^−1^ @ 635 nm, −2.6 × 10^−4^ K^−1^ @ 980 nm), the absolute value of the slope is two orders of magnitude lower [16]. This indicates that novel diamagnetic glass can operate in more complex environments to ensure the stability of high-power laser systems.

## 4. Conclusions

In this paper, we prepared a series of Bi_2_O_3_-Pb_3_(BO_3_)_2_-Ga_2_O_3_-PbO (BP) bismuthate glasses with a gradient change in PbO content, and investigated the changes in their physical, structural, and magnetic properties and Verdet constant. The results indicate that increasing the PbO content can effectively adjust the optical band gap of the glass from 2.62 eV to 2.48 eV. While promoting the conversion of bismuth from Bi^3+^ to Bi^5+^, it also increases the content of non-bridging oxygen in the glass. These structural changes ultimately lead to a decrease in the glass’s thermal stability and a reduction in the nanosecond laser-induced damage threshold (LIDT) from 12.61 J/cm^2^ to 8.33 J/cm^2^. The hysteresis loops of the glasses indicate that all samples exhibit typical diamagnetic characteristics, with their diamagnetic susceptibilities increasing from −11.55 × 10^−6^ emu/g to −13.32 × 10^−6^ emu/g as the PbO content rises. Under the premise of ensuring the stability of the glass, the Verdet constant of BP-4 glass at 633 nm can reach −0.191 min·G^−1^·cm^−1^, which is higher than some tellurite glass and bismuthate glass. In addition, the variation in the bismuthate glasses’ Verdet constant at 1064 nm within 20 and 80 °C is less than 0.4 × 10^−5^ K^−1^, which indicates that these bismuthate glasses are good candidates for magneto-optical devices under thermally unstable conditions.

## Figures and Tables

**Table 1:** Information on the source and purity of the raw materials.

Chemical Formula	Purity	Manufacturer	Country
Bi_2_O_3_	99.99%	Alfa Aesar (Shanghai)	China
Pb_3_(BO_3_)_2_	99.00%	DaXiao Chemical (Guangdong)	China
Ga_2_O_3_	99.99%	Alfa Aesar (Shanghai)	China
PbO	99.99%	Alfa Aesar (Shanghai)	China

**Table 2:** Physical properties of the series of bismuthate glasses.

	ρ (g/cm^3^)	n_d_	ε	Λ_th_	α_O_^2−^	R_m_	V_m_ (cm^3^)	OPD
BP-1	7.787	2.150	4.623	0.96	2.35	20.51	37.49	57.62
BP-2	7.898	2.192	4.805	0.98	2.41	21.49	38.44	57.44
BP-3	8.057	2.242	5.027	1.00	2.49	22.60	39.44	57.45
BP-4	8.250	2.304	5.308	1.03	2.59	23.97	40.65	57.52

**Table 3:** Deconvolution fitting results of high-resolution XPS for the bismuthate glasses.

	**O 1s (1)**		**O 1s (2)**		**(1)/(2)**
	**BE (eV)**	**Area**	**BE (eV)**	**Area**	
BP-1	529.4	20.93	530.6	36.59	0.26
BP-2	529.4	22.42	530.6	77.58	0.29
BP-3	529.3	23.20	530.5	73.29	0.30
BP-4	529.3	24.83	530.5	46.33	0.33
	**Bi 4f _7/2_ (1)**		**Bi 4f _7/2_ (2)**		**(1)/(2)**
	**BE (eV)**	**Area**	**BE (eV)**	**Area**	
BP-1	158.7	48.78	159.1	8.36	5.83
BP-2	158.7	48.26	158.9	8.89	5.43
BP-3	158.7	45.97	158.9	11.17	4.12
BP-4	158.7	44.63	159.3	12.51	3.57
	**Pb 4f _7/2_ (1)**		**Pb 4f _7/2_ (2)**		**(1)/(2)**
	**BE (eV)**	**Area**	**BE (eV)**	**Area**	
BP-1	138.0	22.70	138.2	34.44	0.66
BP-2	138.0	23.69	138.2	33.45	0.71
BP-3	138.0	25.24	138.2	31.90	0.79
BP-4	138.0	28.61	138.5	29.53	0.97
	**Ga 2p _3/2_ (1)**		**Ga 2p _1/2_ (2)**		**(1)/(2)**
	**BE (eV)**	**Area**	**BE (eV)**	**Area**	
BP-1	1117.7	60.01	1144.6	39.99	1.50
BP-2	1117.7	59.96	1144.6	40.04	1.50
BP-3	1117.6	60.31	1144.5	39.69	1.52
BP-4	1117.7	60.12	1144.6	39.88	1.51

**Table 4:** Magnetic susceptibility (χ) and Verdet constant (V) of the series of bismuthate glasses.

	χ (×10^−6^ emu/g)	V (min·G^−1^·cm^−1^)			
		532 nm	633 nm	808 nm	1064 nm
BP-1	−11.55	−0.307	−0.166	−0.078	−0.039
BP-2	−12.21	−0.324	−0.172	−0.090	−0.044
BP-3	−12.49	−0.343	−0.180	−0.097	−0.049
BP-4	−13.32	−0.376	−0.191	−0.106	−0.056

**Table 5:** Verdet constant of diamagnetic oxide glasses from the literature.

Glass	Wavelength (nm)	V (min·G^−1^·cm^−1^)	Ref.
TeO_2_-WO_3_-PbO	633	−0.11	[15]
Bi_2_O_3_-B_2_O_3_-Ga_2_O_3_-TiO_2_	633	−0.163	[36]
Bi_2_O_3_-PbO-B_2_O_3_-ZnO-Sb_2_O_3_	633	−0.121	[42]
Bi_2_O_3_-PbO-GeO_2_-B_2_O_3_	633	−0.162	[43]
Fused SiO_2_	635	−0.013	[41]
TeO_2_-ZnO-BaO	633	−0.113	[44]
TeO_2_-WO_3_-PbO-La_2_O_3_	600	−0.065	[45]
SF57	633	−0.077	[46]
BP-4	633	−0.191	This work

**Table 6:** Verdet constant of the bismuthate glass samples at 1064 nm under different temperatures.

Temperature (°C)	V (min·G^−1^·cm^−1^)			
	BP-1	BP-2	BP-3	BP-4
20	−0.039	−0.044	−0.049	−0.056
30	−0.039	−0.044	−0.049	−0.055
40	−0.038	−0.043	−0.048	−0.055
50	−0.038	−0.043	−0.047	−0.054
60	−0.037	−0.042	−0.047	−0.054
70	−0.037	−0.041	−0.047	−0.053

## Data Availability

The original contributions presented in this study are included in the article. Further inquiries can be directed to the corresponding author.
